# Systematic Review of Phylogenetic Analysis Techniques for RNA Viruses Using Bioinformatics

**DOI:** 10.3390/ijms26052180

**Published:** 2025-02-28

**Authors:** Irena Wadas, Inês Domingues

**Affiliations:** 1 Polytechnic University of Coimbra, Rua da Misericórdia, Lagar dos Cortiços, S. Martinho do Bispo, 3045-093 Coimbra, Portugal; a2024106799@esac.pt; 2 Research Centre of the Portuguese Institute of Oncology of Porto (CI-IPOP), Medical Physics, Radiobiology and Radiological Protection Group, R. Dr. António Bernardino de Almeida, 4200-072 Porto, Portugal

**Keywords:** phylogenetic analysis, bioinformatics, RNA viruses

## Abstract

The present paper addresses topics from various fields of biology. Its purpose is to enlarge the understanding of the usage of bioinformatics tools in the phylogenetic analysis of RNA viruses. The paper highlights the benefits of using information technology in virology, bringing the scientific community closer to unraveling the mysteries of RNA virus evolution and their adaptation to different niches and hosts and facilitating the understanding of their rapid mutation processes. Phylogenetic analysis of genetic sequences allows the exploration of the causes of these genetic changes in viruses and categorizes them into taxonomic groups. This paper is a systematic review of the most important scientific articles on the phylogenetic analysis of RNA viruses using bioinformatics. The studies included in the review were selected based on the Preferred Reporting Items for Systematic Reviews and Meta-Analyses (PRISMA 2020) guidelines and discuss methods for analyzing genetic and protein sequences (including codon sequences) and describe phylogenetic analyses and the bioinformatics tools used (such as VConTACT, RAxML, etc.). This review emphasizes the importance of further development in the fields of bioinformatics and virology, particularly with respect to RNA viruses, in order to mitigate the risk of a future pandemic. It also aims to provide a detailed understanding of the mutation and evolution mechanisms of these entities, which will help in efforts to limit viral virulence, for example. This article did not receive any funding for its creation and has not been registered in any database.

## 1. Introduction

This paper is a review of existing scientific articles on the phylogenetic analysis of RiboNucleic Acid (RNA) viruses using bioinformatics. Phylogenetic analysis is a fundamental research method in genetics and molecular biology, enabling the reconstruction of the evolutionary history of organisms or genes based on their similarities and differences in RNA, DNA, or protein sequences. Its primary goal is to determine the relationships between various species, strains, or genes, allowing for the tracing of their shared evolutionary history. The results of such analyses are typically presented as a phylogenetic tree, a graphical representation of evolutionary relationships. This tree consists of branches, which symbolize evolutionary lineages leading to modern species or sequences, and nodes, which represent common ancestors.

In the context of RNA viruses, phylogenetic analysis facilitates their classification, assessment of relationships, and placement within the phylogenetic tree. This allows for the discovery of new strains or species and their assignment to appropriate families, aiding in the understanding of genome variability and viral evolution. This variability, inherent to the genetic material of RNA viruses, can influence their ability to evade host defense mechanisms, thereby increasing their pathogenicity and potential to cause epidemics or pandemics.

**Figure 1:**
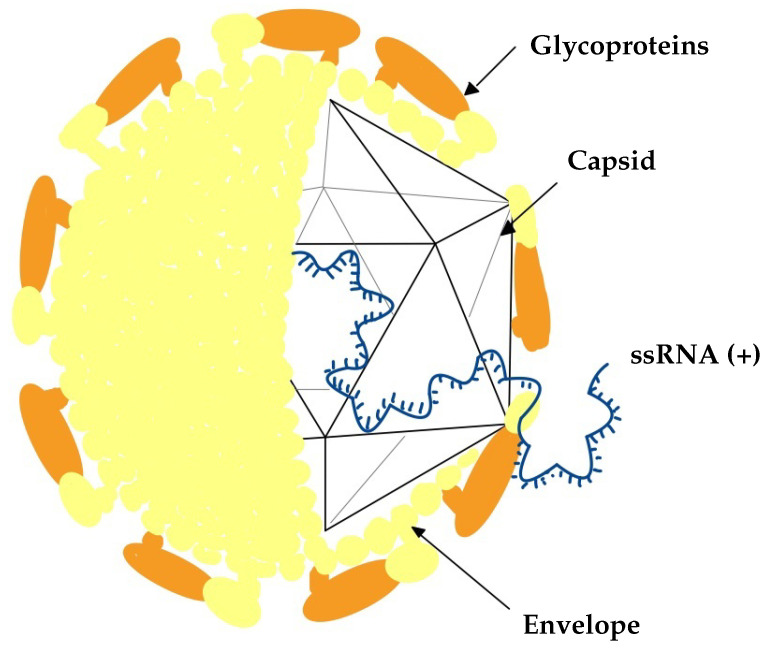
Scheme of HCV virus as an example, inspired by [1]. The enveloped HCV virion includes glycoproteins, while the viral genome consists of a single positive-sense RNA molecule, ssRNA (+), enclosed within an icosahedral capsid formed by core proteins.

Viruses are microscopic infectious particles composed of genetic material (RNA or DNA) enclosed in a protein shell known as a capsid. Some viruses also possess a lipid envelope. This is shown in Figure 1 to simplify and demonstrate an example of the schematic structure of an RNA virus, using the HCV virus as an example. Their replication is possible only within host cells, making them entirely dependent on the host’s biological machinery. The high variability of RNA virus genomes promotes frequent mutations and rapid evolution, further complicating the development of effective treatments or vaccines.

Advanced bioinformatics tools play a crucial role in phylogenetic analysis. They enable the collection, analysis, and comparison of vast amounts of genetic data, accelerating the process of identifying genomic changes in viruses. With the help of databases such as GenBank and bioinformatics software like MEGA, BEAST or MrBayes [2], researchers can identify mutations, compare genetic sequences, and construct phylogenetic trees. These capabilities allow the identification of similarities between viruses, which can support the development of therapies, particularly in cases where related viruses have already been studied.

The creation of this review was motivated by the need to provide a comprehensive overview of the most important bioinformatics methods for analyzing RNA virus genomes, bringing them together in one place. The focus is on demonstrating their applications and how they can facilitate scientific work in classifying newly mutated viruses. Furthermore, the current scientific literature contains relatively few articles that highlight a variety of bioinformatic tools in the context of a limited group of viruses. To date, existing studies tend to either address a broad range of all viruses or focus on a specific group, analyzed using only a limited number of techniques. This review presents a collection of scientific works that gather all the essential elements in one place, filling this informational gap in the existing literature.

In this way, this review aims to provide information on how scientists are looking for this information and how bioinformatics tools enable them to do so. This work will summarize the information and data collected to show the problem, importance, and possibilities of using bioinformatics in the field of analyzing the genetic material of the virus’s RNA. To achieve that goal, the methods are described in Section 2, results are given in Section 3, and a discussion is presented in Section 4.

## 2. Methods

Articles were primarily sourced from Google Scholar, PubMed, Web of Science, and ScienceDirect, with full-text access via platforms like PMC, ScienceDirect, Oxford Academic, and MDPI, during September 2024. Selection was based on the number of citations, recency, relevance to the review’s scope, publication source, focus on viral genomes, bioinformatics tools, and emphasis on viral phylogenetics.

To minimize bias, multiple articles were used as sources, allowing for the identification of repeated information and gaps. When unique information appeared in a single article, additional works on similar topics were consulted to explain discrepancies and validate the findings.

While searching for relevant scientific articles related to the subject of this review, several studies addressing similar topics were encountered. However, the majority of scientific reviews generally focus on viruses as a whole or on the tools used for phylogenetic analysis of various organisms [3]. The present work is one of the few papers that specifically addresses RNA viruses while also describing the devices used for their genomic and phylogenetic analysis. An additional strength of this work is the inclusion of examples that support the theoretical and informational content of this review.

OpenAI was used to translate sources where necessary to prevent the possibility of losing substance in translation and avoid any factual errors. Moreover, it was used in the selection of the most notable tools from the identified sources. More details on the methods can be found in Appendix A.

## 3. Results

This section will focus on explaining the criteria used to determine which parts of the selected articles were excluded from the sources for this review, as well as which articles advanced to further qualification. Another aspect of this section will be to present and describe the most important information, bioinformatics tools, and selected techniques from each article. The next step will involve outlining and drawing appropriate conclusions from the previously presented studies. To enhance the analysis of the information, charts and graphics are included. Additionally, this section will demonstrate the credibility of the conclusions drawn and the results obtained.

### 3.1. Study Selection

To create this paper, material gathering was necessary. For this purpose, the following databases were used: Google Scholar, PubMed, Web of Science, and ScienceDirect. Relevant articles were identified by searching, in the title, abstract, and keywords of papers, the following terms: RNA virus AND phylogenetic analysis AND phylogenetic tree AND bioinformatics tools AND genome AND bioinformatics. The literature search was conducted following the guidelines provided by PRISMA (Preferred Reporting Items for Systematic Reviews and Meta-Analyses) [4] and was limited to articles published between 2010 and 2024. Data extracted from selected articles included the title, publication year, author, and citation count. Subsequently, the research papers were analyzed in depth, focusing on the bioinformatics techniques used, the type of genetic material the virus contained, the virus’s host, its family, and whether a phylogenetic analysis of the RNA virus was present in the article. Information extraction was carried out by the first author, with assistance from Artificial Intelligence.

**Figure 2:**
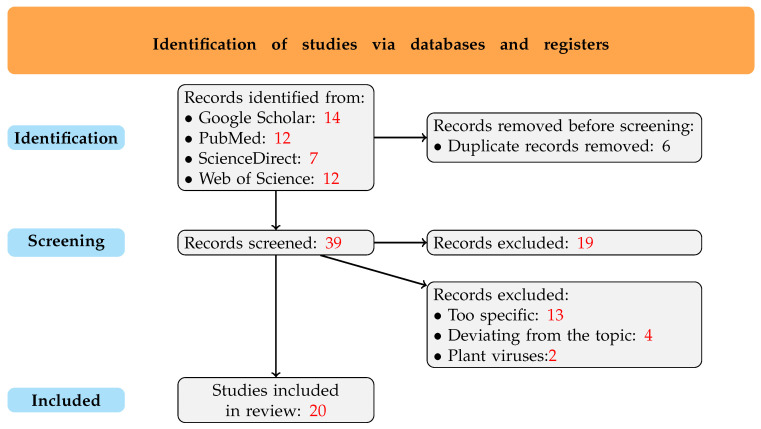
PRISMA flow diagram, outlining the process of article selection.

During the search for articles, a large number of results appeared in the databases. However, after applying filters and analyzing the displayed results, the number of articles was reduced to 45. Among these, six duplicates were identified and removed from the review. Then, after further examining the abstracts and titles of the scientific studies, the following conclusions were drawn: 13 titles were too specific for this review, 4 articles were off-topic, and 2 paper described a plant virus, while the remaining studies focused on viruses infecting animals or humans. In the end, 20 papers were included in the review. The diagram in Figure 2 presents the applied criteria.

The studies referenced to create this review included either the same or alternative bioinformatics techniques for viral genome analysis. These research papers also covered a relatively broad topic area of RNA virus evolution, providing extensive information on phylogenetic analysis. Examples of such studies were “*Bioinformatic Analysis of Codon Usage and Phylogenetic Relationships in Different Genotypes of the Hepatitis C Virus*”, “*Bioinformatics of Virus Taxonomy: Foundations and Tools for Developing Sequence-Based Hierarchical Classification*” [3], and “*Metagenomics Reshapes the Concepts of RNA Virus Evolution by Revealing Extensive Horizontal Virus Transfer*” [5], among others.

However, many articles initially passed the preliminary screening but were ultimately excluded. Reasons for excluding them as information sources for the review included excessive specificity, as seen in “*Discovery, Genomic Sequence Characterization and Phylogenetic Analysis of Novel RNA Viruses in the Turfgrass Pathogenic Colletotrichum spp. in Japan*” [6], “*RdRp-scan: A Bioinformatic Resource to Identify and Annotate Divergent RNA Viruses in Metagenomic Sequence Data*” [7]. Additionally, some articles focused on plant-host viruses, such as “*Detection of RNA Viruses in Cape Gooseberry (Physalis peruviana L.) by RNAseq Using Total RNA and dsRNA Inputs*” [8]. Lastly, another reason for exclusion was an off-topic focus, as in “*Open Reading Frame Phylogenetic Analysis on the Cloud*” [9].

### 3.2. Study Characteristics

The selected articles focus on the application of bioinformatics tools for viral genome analysis to construct phylogenetic trees and understand viral evolution. This process aims to elucidate the relationships among viruses and examine their evolutionary dynamics. The studies used various technologies, including Multiple Sequence Alignment (MSA), which allows the simultaneous comparison of multiple genomic sequences from different viral strains, identifying conserved regions and mutations within the viral genome. This technique serves as a foundational tool for phylogenetic analysis [10].

Metagenomics also aids in constructing phylogenetic trees and understanding viral evolutionary development. This method centers on analyzing viral RNA sequences, enabling researchers to study viruses directly from natural environments without requiring lab cultivation. This approach facilitates the discovery and classification of previously unknown viruses [5,10,11]. Metagenomics has advanced RNA virus phylogenetic tree construction, enabling the identification of previously unknown viral groups. RNA-dependent RNA polymerase (RdRp) sequence analysis, a highly conserved RNA viral gene, has proven invaluable. RdRp is essential for viral replication, and its analysis provides a foundation for tracking viral evolution. Moreover, as an analytical target, RdRp enables the study of relationships between viral families [5].

The key role in studying virus transmission patterns between hosts is played by phylogenetic analysis. It allows for the identification of horizontal gene transfer cases and the investigation of the virus’s evolution. To select an appropriate tool for constructing a phylogenetic tree, prior knowledge of experimental parameters and available resources is needed. Several bioinformatics tools used for phylogenetic trees help in understanding RNA virus adaptation to different hosts. One such tool is MEGA (Molecular Evolutionary Genetics Analysis), which integrates various methods of phylogenetic analysis, assisting users in complex analyses. Additionally, BEAST (Bayesian Evolutionary Analysis Sampling Trees) is best suited for studying epidemic dynamics and temporal analysis, though it requires significant computational power. Meanwhile, metagenomics, by analyzing genetic material without isolating individual organisms, significantly aids in detecting horizontal gene transfer cases between different host species. Environmental sequencing helps to identify viral sequences in different organisms, suggesting cases of interspecies virus transmission. The tools mentioned earlier are used to analyze results from metagenomic studies. By comparing viral sequences from various hosts, we can build phylogenetic trees that show evolutionary links between viral isolates. Examining these trees highlights sequences linked to horizontal virus transfer between host species, providing insights into such viral transmission across species [5].

The viral genome remains a primary resource for virus classification among known viruses, underscoring the critical role of bioinformatics in virology research. Various bioinformatics tools (Table 1) highlighted in the articles include the following:

- PASC: This tool allows rapid viral genome sequence comparison by measuring sequence distance between pairs, crucial for efficiently assigning new viruses within the phylogenetic tree.
- VConTACT: This method leverages genomic and protein structural similarity to group viruses. Primarily used for analyzing bacteriophages and other viruses, it elucidates evolutionary pathways, diversity, and viral similarities. Initially, it compares protein sequences to establish homologs, highlighting common features. A subsequent “network analysis” constructs a schematic network where nodes represent viruses, and connections indicate similarities. Finally, clustering algorithms group viruses with shared features, streamlining classification.
- SDT (Sequence Demarcation Tool): This tool calculates sequence distances, used to delineate species or viral groups. It is critical for viral taxonomy and systematics, assisting in identifying new viruses and understanding their diversity and evolution.
- GRAViTy: This tool supports genetic analysis, identifying common viral domains and enabling sequence-level comparison.

**Figure 3:**
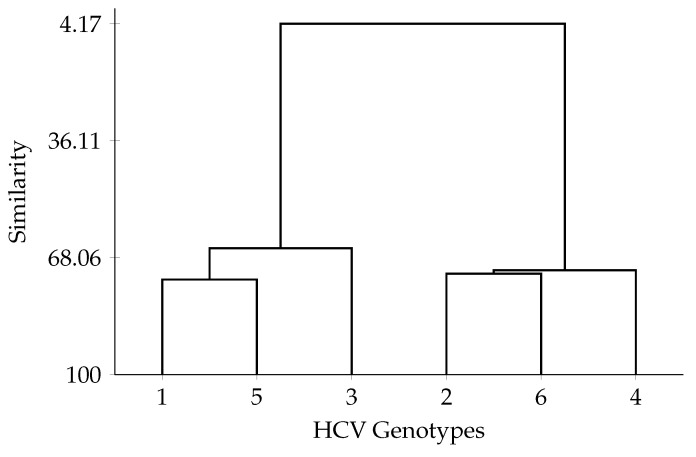
Similarity of codon usage between HCV genotypes. Genotypes are divided into two groups: 1, 5, and 3 in one group, and 2, 6, and 4 in the other group.

Phylogenetic analysis, essential for tree construction, requires genotypic analysis supported by several tools, one of which is “MEGA 7”. This tool enables phylogenetic analysis and evolutionary relationship tracking by using “GenBank” data and the maximum parsimony method, which selects a tree with minimal mutational changes between sequences. This technique is often combined with other methods, such as the Maximum Likelihood method, to capture complex evolutionary patterns and provide more comprehensive genetic comparison material for viral sequences. In the article “*Bioinformatic Analysis of Codon Usage and Phylogenetic Relationships in Different Genotypes of the Hepatitis C Virus*”, these tools were used to compare and understand gene expression mechanisms and protein translation efficiency in HCV. The “Gene Infinity” tool analyzed the frequency of 61 amino acid codons across six HCV genotypes, revealing codon preferences. HCV genotypes favor cytosine (C)-rich and guanine (G)-rich codons, informing mRNA translation processes. Codon analysis enabled RNA virus phylogeny, showing that genotypes 1 and 4 share high protein and genomic sequence similarity despite differing codon usage preferences. This finding indicates evolutionary complexity and diverse codon selection mechanisms within HCV genotypes. Additionally, genotype 1 and genotype 2 are the most distinct in genomic and protein sequences, illustrating HCV diversity (Figure 3).

Constructing a phylogenetic tree requires various tools and methods, including the following:

1. **Sequence analysis tools**: BLAST (Basic Local Alignment Search Tool), crucial for sequence comparison and homolog identification, or Clustal Omega, which provides multiple sequence alignment for assessing viral similarity.
2. **Phylogenetic tree-building software**: MEGA (Molecular Evolutionary Genetics Analysis) supports phylogeny and evolution analysis and enables tree construction using multiple methods (e.g., Maximum Likelihood). PhyML (Phylogenetic Analysis using Maximum Likelihood) is also used to build trees based on the Maximum Likelihood algorithm [3].
3. **Metagenomic data analysis tools**: OiiME analyzes metagenomic data, facilitating viral identification and classification.
4. **Phylogenetic tree visualization tools**: TOL (Interactive Tree of Life) allows interactive tree visualization and the addition of essential information to tree schematics.

**Figure 4:**
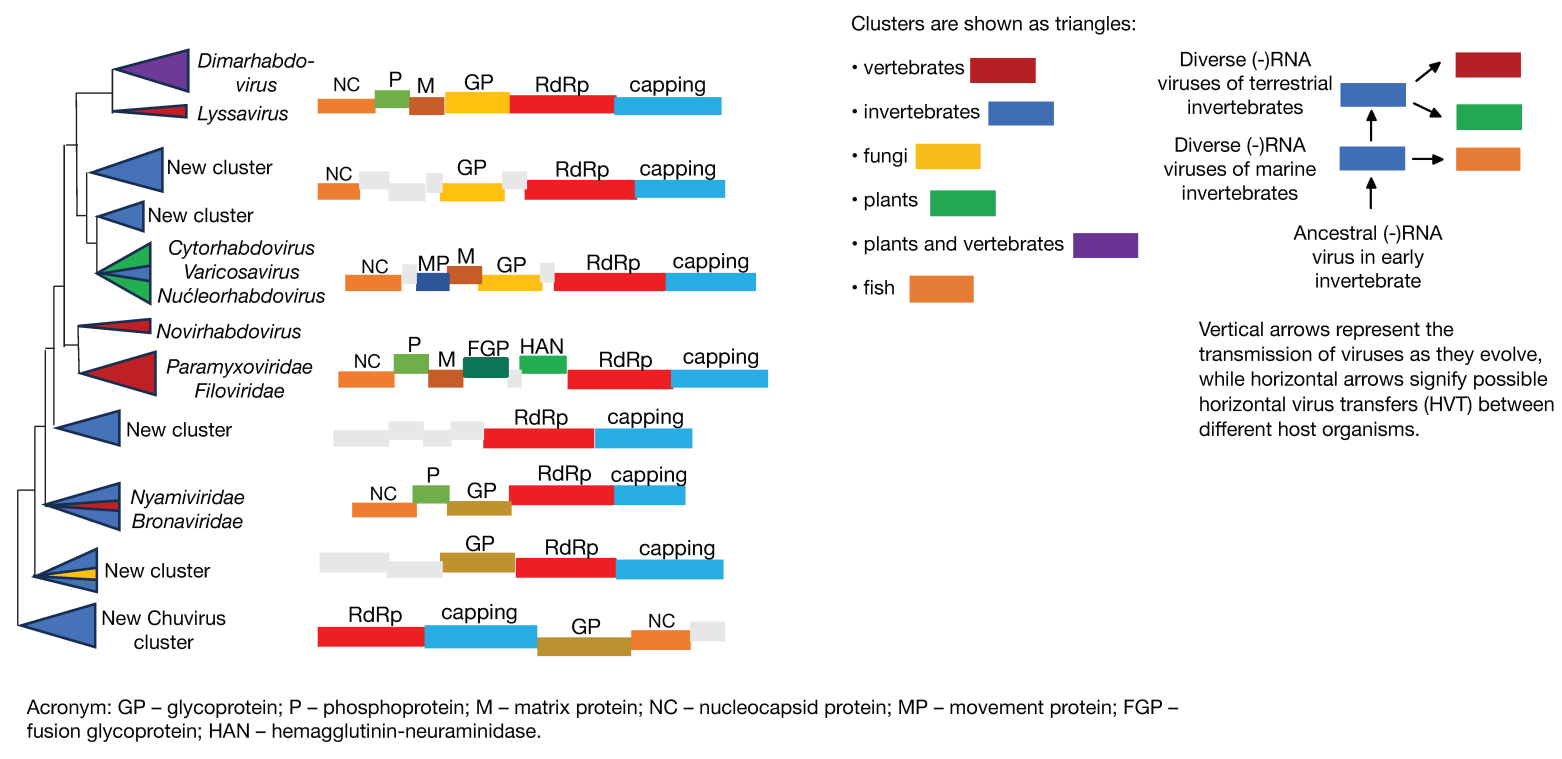
A schematic phylogenetic tree illustrating the hypothetical occurrence of horizontal virus transfer (HVT) from the viromes of ancestral organisms to newly emerging groups of viruses, own interpretation influenced by [5].

These tools are commonly combined for phylogenetic tree building and visualization. In the article “*Metagenomics reshapes the concepts of RNA virus evolution by revealing extensive horizontal virus transfer*” [5], these methods illustrated Horizontal Gene Transfer (HGT), whereby viruses exchange genetic material independently of evolutionary lineages (Figure 4).

The schematic in Figure 4 highlights the evolutionary pathway of negative-sense RNA viruses, highlighting both horizontal gene transfer and vertical transmission. It depicts various virus families, including *Nyamiviridae, Bronaviridae, Paramyxoviridae, Filoviridae* and different groups of *rhabdoviruses*. The classification is based on their hosts, which include vertebrates, invertebrates, fungi, plants, and fish. Horizontal virus transfer between different organisms indicates their ability to adapt and generate new variants, represented as newly identified clusters. These clusters suggest the discovery of novel evolutionary lineages. This phylogenetic tree indicates that RNA virus evolution is more dynamic and complex than previously assumed. HGT may promote the development of new strains, increasing pathogenicity and viral transmission capability.

In the article “*Coronavirus Genomics and Bioinformatics Analysis*” [10], several key bioinformatics tools were used to trace the evolutionary trajectory of the coronavirus genome and explain the relationships between its strains. Multiple Sequence Alignment (MSA) was employed to gather data for constructing a phylogenetic tree, which illustrated the relatedness of various coronavirus strains through the analysis of conserved genome regions, specifically the RNA-dependent RNA polymerase gene and the nucleocapsid protein gene. This approach allowed the identification of genetic distinctions among strains and clarified their evolutionary relationships.

Additionally, a molecular clock was implemented to monitor evolutionary rates based on gene mutation patterns, enabling the estimation of the time when different strains shared a common ancestor. This tool provided insight into the virus’s evolutionary pathway and its dissemination. Evolutionary pressures on the coronavirus were assessed using the molecular clock and gene sequence analysis of ORF1ab (which comprises two-thirds of the genome and encodes replication polyproteins), spike (S) protein (responsible for viral entry into host cells and a highly variable coronavirus protein), and nucleocapsid (N) protein (valuable in phylogenetic studies due to its conserved nature across coronaviruses). This analysis revealed how various coronavirus clades (alpha, beta, gamma) evolved and adapted to different hosts.

**Figure 5:**
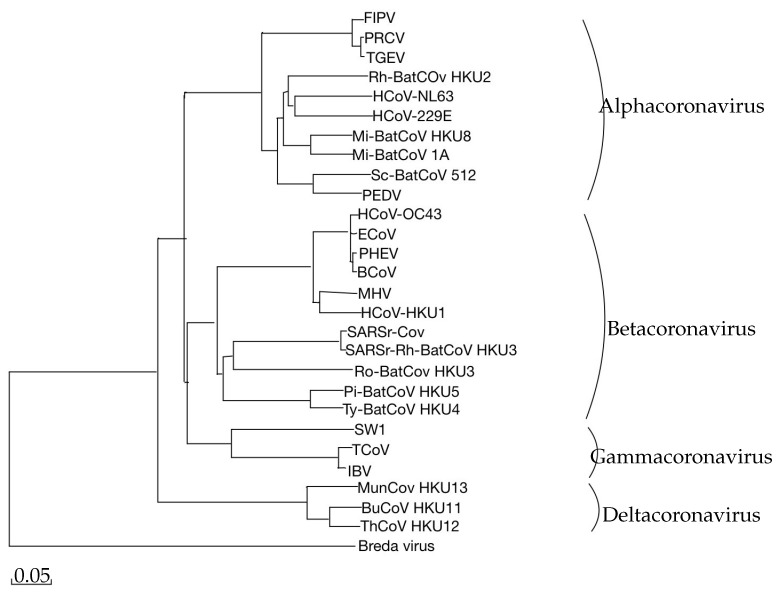
Phylogenetic analysis of RNA-dependent RNA polymerases (Pol) of coronaviruses with complete genome sequence, based on [10]. The scale bar represents the estimated number of substitutions occurring per 20 amino acids.

A crucial method employed in this study was Codon Usage and Mutation Analysis, which facilitates the investigation of codon usage patterns and the effects of mutations on genome composition. This technique aids in identifying the evolutionary forces driving coronavirus genomic diversity, such as cytosine deamination or CpG site selection pressures (Figure 5) [8].

The primary branches of the phylogenetic tree encompass four main strains:

- *Alphacoronavirus*—includes viruses predominantly infecting mammals;
- *Betacoronavirus*—comprises various subgroups, including significant strains such as SARS-CoV, MERS-CoV, and SARS-CoV-2;
- *Gammacoronavirus*—primarily contains avian viruses;
- *Deltacoronavirus*—includes both avian and human viruses.

Furthermore, the RdRp analysis allows for the determination of the evolutionary proximity among the listed strains and coronavirus species.

In the recent selected article [6], a novel sequencing method called FLDS (Fragmented and Primer-Ligated dsRNA Sequencing) is introduced. This method allows for the identification and sequencing of multi-segmented viral genes without prior knowledge of their structure. This was particularly significant in this case, as the study focused on a new RNA virus from the fungi *Colletotrichum*.

To complete this research, a bioinformatics analysis was required, involving several techniques:

- **CLC Genomics Workbench**: used to assemble sequences from FLDS reads.
- **BLASTn and BLAST**: necessary to compare the sequences of the capsid protein and RNA-dependent RNA polymerase with those of other known viruses.
- **IQ-TREE**: used to construct a phylogenetic tree using a Maximum Likelihood algorithm.

The results of the study led to the identification of four new viruses, two of which are double-stranded RNA viruses, classified as new species within the family *Partitiviridae*. The other two, single-stranded RNA viruses, were assigned to the family *Phenuiviridae*. These conclusions were reached through phylogenetic analysis focusing on the sequences of the RdRp and capsid proteins of the isolated viruses.

However, the primary challenge for these tools is posed by the rapid evolution of RNA viruses through mutations or recombination, which may necessitate frequent updates to algorithms and databases to reflect the current state of knowledge. Another significant issue lies in the analysis of HGT, as these tools do not always effectively capture the dynamics of this process, which is critical to understanding the evolution of RNA viruses.

#### The Impact of Mutational Tendencies on RNA Virus Phylogeny

Genetic changes in the RNA virus group are primarily driven by point mutations, recombination, and horizontal gene transfer. However, in this type of virus, base mutations mainly occur due to errors made by RNA-dependent RNA polymerase (RdRp). Since RdRp lacks a proofreading mechanism, these mutations are often random. Consequently, base mutations in RNA viruses are not caused by error-prone repair but rather by the absence of proofreading in RdRp, random replication errors, and recombination, which form the foundation of every phylogenetic tree [6].

In every virus, base sequence mutations occur, leading to further evolutionary tendencies. This has a significant impact on the evolution and evolutionary trajectory of these entities, which, in turn, is reflected in phylogenetic trees and the phylogenetic analysis of RNA viruses. The presence of such tendencies influences phylogenetic trees through:

- **Mutational preferences**: This group of viruses exhibits tendencies toward specific types of mutations, such as purine-to-purine or pyrimidine-to-pyrimidine substitutions. These changes can lead to an uneven distribution of mutations across the RNA genome, which in turn affects the shape and interpretation of the phylogenetic tree.
- **Structural effects**: Various mutations can impact the structure of viral proteins, influencing their function and interactions with the host. Such changes may enable viruses to adapt to new hosts or ecological niches. This adaptation is reflected in phylogenetic trees as newly emerging clades or branches.
- **Positive and negative selection**: Natural selection can either favor or eliminate certain mutations, altering the direction and pace of viral evolution. Beneficial mutations lead to faster branching of lineages within the phylogenetic tree, while disadvantageous mutations may be rapidly removed, resulting in shorter branches in the tree.

To observe these tendencies in phylogenetic analyses, it is essential to use evolutionary models that account for mutational biases. These models highlight specific mutational preferences, facilitating a more precise reconstruction of the evolutionary history of the virus. Additionally, during research, it is useful to analyze the impact of selection by identifying genome regions subjected to strong positive or negative selection. This can help in understanding the evolutionary dynamics of the virus and its influence on the structure of the phylogenetic tree.

Mutational tendencies in base sequences, along with the associated selection processes, play a crucial role in shaping and interpreting RNA virus phylogenetic trees. Incorporating these factors into phylogenetic analyses significantly enhances our understanding of the evolutionary processes governing these viral particles [12].

Mutations in the amino acid sequence play an important role in determining a virus’s potential functions. Additionally, genetic changes can affect cis-regulatory elements, such as promoters, signal sequences, and enhancers, which restrict, silence, or initiate gene expression and viral replication. Accounting for changes in the amino acid sequences of cis-elements in phylogenetic analyses can thus enrich our understanding of viral evolution and function. Several methods exist for incorporating changes in cis-elements into phylogenetic studies. These include analyzing noncoding sequences, developing evolutionary models that integrate alterations in both coding and noncoding regions, and functionally analyzing mutations in these elements. While basic phylogenetic analysis focuses on protein-coding sequences, examining nonprotein-coding regions (for example, promoter regions) enables the identification of conserved motifs that influence gene expression. Comparing these regions across different strains or species accelerates and simplifies the detection of evolutionary modifications in gene regulation.

Moreover, the development of evolutionary models that combine changes in coding and noncoding sequences allows for a more detailed analysis, revealing differences in the evolutionary rates of specific regions and their impact on viral function. Experimental functional studies, such as promoter activity assays or transcription factor binding analyses, help determine the effects of specific mutations in cis-elements on gene expression, and when combined with phylogenetic analyses, they facilitate a deeper understanding of adaptive changes in gene regulation.

Recent research has increasingly highlighted the importance of regulatory sequences in viral evolution. For instance, an analysis of HIV-1 demonstrated that a mutation in the LTR promoter region can affect both the replication timing and infectivity of the virus. Phylogenetic studies that incorporate key regulatory regions and cis-elements can thus provide additional insights into viral evolution and the adaptation of viruses to their environments and hosts [13].

Reversion mutations are reverse mutations that occur in the genomes of RNA viruses. They involve substituting one nucleotide base for another, effectively reversing a previous mutation. In constructing phylogenetic trees, these mutations can affect the specificity of the analysis and may lead to erroneous conclusions.

Traditional phylogenetic algorithms rely on analyzing RNA or DNA sequences, and reverse nucleotide mutations can contribute to homoplasy, a situation where similarities between sequences result from convergent evolution rather than a true common ancestry. This, in turn, can result in inaccurate phylogenetic trees. Modern phylogenetic methods strive to account for reversion mutations in order to produce the most accurate and reliable trees possible. However, due to the complexity of evolutionary processes, completely eliminating the influence of nucleotide reversion mutations is challenging. To minimize the impact of such mutations, various strategies are employed. One approach is to use appropriate molecular evolution models that incorporate different types of mutations, including reversions. An example is the General Time Reversible (GTR) model, which accounts for all possible substitutions between nucleotides, including reverse mutations. Models of this kind are implemented in programs such as RAxML and IQ-TREE. Another method involves using tools to analyze homoplasy. Bioinformatics techniques that identify and minimize the effects of homoplasy on phylogenetic tree construction can be invaluable. The application of Bayesian inference algorithms, as implemented in MrBayes, can help eliminate errors associated with homoplasy, including those caused by reversion mutations [14,15].

In conclusion, reversion and homoplasy processes have a significant impact on the construction of phylogenetic trees, particularly for RNA viruses. However, the use of appropriate algorithms, such as Bayesian inference, together with evolution models that account for reversions (e.g., GTR), helps minimize the influence of these mutations on analytical outcomes.

### 3.3. Risk of Bias in Studies

Each study was carefully analyzed and selected to ensure that the techniques used were similar, while varying in specific aspects, such as the algorithms employed in the bioinformatics analyses. Additionally, each study focused on a different type of virus, minimizing the potential for bias. Furthermore, all the papers drew similar conclusions, although corresponding to their specific topics.

All articles shared comparable assumptions regarding phylogenetic analysis and the examination of RNA viral genomic sequences, leading to the conclusion that each study was conducted appropriately, with minimal risk of bias. This conclusion is further supported by the fact that the studies were published in reputable databases and scientific journals. Moreover, each paper used as a source has been cited by other researchers in the scientific community.

### 3.4. Results of Syntheses

The article “*Bioinformatic Analysis of Codon Usage and Phylogenetic Relationships in Different Genotypes of the Hepatitis C Virus*” is based on an analysis of RNA virus codons using bioinformatics tools to construct a phylogenetic tree among different genotypes of the hepatitis C virus (HCV). This analysis highlights the similarities and differences between these genotypes, as well as their evolutionary trajectories. Furthermore, the study incorporates phylogenetic relationship analysis to refine the results. Genotypes 1 and 4 of HCV show the greatest genomic and protein sequence similarity, although they exhibit distinct codon usage preferences. Additionally, genotypes 1 and 2 display the highest genomic sequence divergence, underscoring the extensive variability of the HCV virus.

The study titled “*Bioinformatics of Virus Taxonomy: Foundations and Tools for Developing Sequence-Based Hierarchical Classification*” [3] provides valuable insights into the use of various bioinformatics tools for classifying and organizing RNA viruses based on their genomic sequences. With the growing accessibility of advanced genome sequencing techniques, bioinformatics tools are becoming foundational for constructing phylogenetic trees that illustrate the evolutionary paths of these viruses. This article introduces several helpful tools, methods, and techniques that facilitate laboratory work, including VConTACT, SDT, PhyML, and GRAViTY. The paper highlights the crucial role of these tools in virus classification, which enhances the understanding of viral evolution and aids in the identification of new viruses and their taxonomic groups.

The next article, “*Metagenomics Reshapes the Concepts of RNA Virus Evolution by Revealing Extensive Horizontal Virus Transfer*” [5], proved to be the most crucial for preparing this review. It places special emphasis on a key factor in RNA virus evolution: horizontal virus transfer. This study shows that viral horizontal transfer can occur between different host groups, such as plants, animals, and protists. It also concludes that viruses have repeatedly migrated from invertebrates (such as arthropods and nematodes) to plants and vertebrates through horizontal transfer. This research draws attention to metagenomics, which has expanded the phylogenetic trees for RNA viruses, revolutionizing our understanding of how RNA viruses may migrate and evolve across various ecological niches. Additionally, through the use of other bioinformatics tools—such as BLAST, MAFFT, and ClustalW—for comparing RNA-dependent RNA polymerase genetic sequences and constructing phylogenetic trees, the study enabled the discovery of new superfamilies of small, positive-sense RNA viruses.

All the subsequent articles contributed equally important information to the review. The research paper titled “*Challenges in RNA Virus Bioinformatics*” [11] presents the tools used for analyzing RNA viruses, focusing on sequence assembly, structure prediction, and virus classification. A particularly enlightening aspect of this work was the emphasis on RNA structure prediction using tools such as “RNAz” and “evofold”. These tools help identify conserved secondary RNA structures, which play a significant role in understanding RNA functionality within viral genomes. Based on these structures, cis-regulatory elements that control viral replication and gene expression emerge. Additionally, this article, like the previous ones, underscores the importance of phylogenetic analysis using tools such as “BEAST” and “RAxML”. This technique facilitates the construction of phylogenetic trees and enhances our understanding of evolutionary relationships and diversity within RNA virus families. This methodological approach is valuable as it can accommodate the rapid mutation and recombination rates typical of viruses. The research highlights that this field still requires further development to gain a more precise understanding of virus–host interactions and to aid in monitoring viral resistance to treatments and the effectiveness of vaccines.

The next article, “*Coronavirus Genomics and Bioinformatics Analysis*”, ref. [10], provides a detailed overview of coronavirus genomes, focusing on the use of bioinformatics tools for analyzing the genome and the evolutionary dynamics of the RNA virus, as well as constructing phylogenetic trees. This study has facilitated an understanding of the relationships among different virus types and has illustrated the evolution and diversity of coronavirus strains, which is crucial for tracking potential pandemic threats, especially in light of recent events. This article serves as a valuable source of information, demonstrating that research on viruses can be a preventive measure against large-scale tragedies.

The final article included in this review, “*Discovery, Genomic Sequence Characterization and Phylogenetic Analysis of Novel RNA Viruses in the Turfgrass Pathogenic Colletotrichum* spp. *in Japan*” [6], used bioinformatics tools to discover new viral families in fungi. Four novel viruses were identified: two are single-stranded RNA viruses with negative polarity, while the other two possess double-stranded RNA. The newly discovered pathogenic species have been assigned to the families “Partitiviridae” and “Phenuiviridae”, with a proposed family name of “Mycoaspirividae”. Through their discovery and phylogenetic analysis, which demonstrated their relationship to other fungi-infecting viruses, conclusions were drawn regarding their potential adaptability and ability to infect various host types. Mycoviruses have the potential to weaken pathogenic fungi, and these newly identified viruses could play a crucial role as biological control agents against pathogens.

Each article selected for this scientific review was carefully chosen to ensure that the information presented in each one complements the others while conveying a similar overall message. All scientific papers used were thoroughly analyzed multiple times, both before and during the writing process, to mitigate the risk of bias. This claim is supported by several arguments, including the fact that each article was sourced from reputable databases, has been frequently cited, and has undergone scrutiny and evaluation by members of the scientific community. Any missing results or misinterpretations within the selected studies would likely have been identified by other authors referencing these works, as well as by the authors of this review. Thus, it can be concluded that the research papers included in this review are free from bias and other significant errors that could undermine their credibility.

During the preparation of this review of scientific papers, no statistical synthesis was conducted. Additionally, a meta-analysis was not performed because this article is a collection of scientific works aimed at extracting as much useful information and data as possible. The purpose of this review was not to conduct any laboratory work, so there were no results available for mathematical or statistical analysis. Consequently, presenting the results of a statistical synthesis are not feasible.

Among the selected scientific studies, no significant ambiguities emerged. This may be attributed to the careful selection of articles with similar themes. Each study reached similar conclusions and employed bioinformatics tools for the same purposes, although they focused on different groups of RNA viruses. The only noticeable difference between the works is the conclusion that bioinformatics tools and techniques require further development. This assertion is crucial and is supported by compelling arguments. It is driven by the rapid evolution and development of RNA viruses, which can be pathogenic to humans and animals, representing a significant factor that could lead to pandemics. Additionally, each article concluded that phylogenetic analysis and bioinformatics techniques greatly facilitate understanding of viruses, their characterization, identification, and classification into appropriate taxonomic groups, thereby illustrating the relationships among them.

### 3.5. Reporting Biases

During the writing of the review, multiple analyses and searches for specific information were conducted. However, no noticeable absence of results or data was encountered in any of the studies. In the selected articles, there were no doubts raised regarding the findings, nor was there any emphasis on poorly conducted research. These factors contributed to nearly 100% confidence in the absence of bias risks stemming from missing results in the chosen studies.

### 3.6. Certainty of Evidence

All information, conclusions, and results presented in this review have been derived from approved articles as sources. This suggests that the data presented in this work are reliable, as they are based on highly cited studies published in reputable scientific journals. Additionally, this review has undergone multiple revisions and analyses alongside selected scientific studies. In referencing these studies, the author has verified their credibility and presence in several respected databases. All of these factors provide a solid foundation for confidence in the validity of the conclusions drawn.

## 4. Discussion

This section presents a summary of studies analyzing RNA viruses, including HCV and coronaviruses, using bioinformatics tools such as MEGA 7, VConTACT, and GRAViTY. The key findings provide insights into viral taxonomy, evolutionary pathways, and codon preferences, which are crucial for understanding the rapid adaptation of viruses and developing various medical solutions, such as antiviral therapies. Additionally, the significant role of metagenomics is highlighted, as it has enabled the discovery of diverse and previously unknown virus species. Meanwhile, research on horizontal transfer and RNA recombination emphasizes their important roles in viral evolution and pandemic risk. Combining metagenomic and bioinformatics approaches facilitates monitoring and counteracting emerging threats related to RNA virus development.

### 4.1. General Interpretation

All articles gathered for this review focus on the analysis of RNA viruses, including HCV and coronaviruses, using bioinformatic tools to study virus taxonomy and evolutionary pathways. The selected papers employed various bioinformatic methods, such as MEGA 7, VConTACT, GRAViTY, and codon analysis tools. These approaches allowed for the creation of complex phylogenetic trees and provided insights into the codon preferences of specific virus types. This research, among other findings, has enabled scientists to gain a clearer understanding of the evolutionary relationships between different HCV genotypes and explore mechanisms used by viruses to adapt to new environments.

### 4.2. Limitations of the Review

As presented in the HCV study, codon analysis plays a significant role in identifying which codons are favored by the virus. Viral codon preferences indicate the efficiency of protein translation of these viruses. The study suggests that codon preferences may be crucial for medical applications, such as developing personalized antiviral therapies. Additionally, codon analysis highlighted that HCV genotypes 1 and 4, despite their genomic similarity, exhibit distinct codon preferences, suggesting that these viruses demonstrate evolutionary complexity where environmental selection plays a substantial role in their adaptation.

The classification of RNA viruses has become increasingly accessible through bioinformatic tools like VConTACT and PASC, which enable rapid and efficient analysis of genomic sequences [11]. Additionally, the use of metagenomics in analyzing RNA viruses, such as coronaviruses, has modernized our understanding of the diversity and evolution of these viruses. This technique allows for the study of viruses without the need for prior cultivation, thereby facilitating the discovery of genetic diversity directly in natural environments and the identification of previously unknown viruses. A significant advantage of this method is also the ability to analyze interactions between viruses and other microorganisms [5].

To better understand the dynamics and mechanisms of virus transmission across different host types, it is essential to consider horizontal transfer in RNA viruses and its impact on viral evolution. Horizontal virus transfer can lead to the emergence of new strains, illustrating how intricate and multi-faceted the evolutionary process of viruses is [5].

In addition, homologous RNA recombination, which can result in new viral genotypes, should be closely monitored, as it represents a significant threat in the context of potential pandemics, as evidenced by genomic analyses of coronaviruses. The growing number of identified coronavirus strains, thanks to advancements in bioinformatics and the expansion of databases such as CoVDB, allows for more precise phylogenetic analyses and the monitoring of viral adaptation to new environments or hosts [10].

### 4.3. Limitations of the Bioinformatic Tools

The disadvantages and limitations of the bioinformatics tools discussed in the article can be categorized according to technological and algorithmic constraints. Tools such as VConTACT and PASC exhibit high specificity and are optimized for certain types of viruses, limiting their applicability for broader analyses. In addition, metagenomics requires high-quality data, and when results with limited accuracy are obtained, sequencing errors can arise, potentially leading to false conclusions. Similarly, tools such as MEGA 7 and PhyML struggle to detect subtle differences in the evolution of viruses with highly conserved genes.

To address some of these limitations, network analysis has emerged as a complementary approach to traditional phylogenetic methods. Unlike standard phylogenetic trees, which assume a strictly branching evolutionary pattern, network analysis can incorporate more complex evolutionary processes such as recombination and horizontal gene transfer (HGT). This capability provides a more accurate representation of RNA virus evolution, particularly for highly variable viruses.

Network analysis enables the identification of recombination events, the tracking of intricate transmission pathways, and much more. For instance, studies on SARS-CoV-2 and tick-borne encephalitis virus (TBEV) have used network analysis to map complex relationships between viral strains and to detect recombination events, providing detailed insights into the dynamics of RNA virus evolution and transmission [16].

Moreover, specialized network analysis tools are employed in these studies. One such tool is GARD (Genetic Algorithm for Recombination Detection), which is used to identify regions of recombination in RNA sequences. Another tool, PopART (Population Analysis with Reticulate Trees), creates phylogenetic networks that better represent complex evolutionary relationships, including recombination events. An especially interesting tool is HIV-TRACE, originally designed for analyzing HIV transmission networks but later adapted for other RNA viruses [16,17].

Beyond network-based approaches, advanced computational methods are currently being developed that combine the prediction of three-dimensional protein structures and functions with phylogenetic analyses, enabling a deeper understanding of protein evolution and biological roles, including those of viral proteins. One approach involves the analysis of coevolution among amino acid residues, based on the premise that residues in close proximity within a protein’s structure coevolve to maintain its shape and function. Investigating co-variation patterns in homologous sequences facilitates the prediction of contacts between residues, allowing for the modeling of three-dimensional structures using maximum entropy schemes. Another method employs phylogenetic profiles, which examine the presence or absence of specific genes or protein domains across various organisms, thereby providing insights into protein function, interactions, and cellular localization. A subsequent step involves integrating structural data with phylogenetic information. This integration permits the study of changes in the three-dimensional structure of proteins within the context of their evolutionary history, as well as the identification of key residues responsible for function and adaptation to environmental conditions. Applications of these methods include de novo protein structure prediction enabling the modeling of structures without available experimental data and the identification of functions for proteins with unknown structures through the analysis of their occurrence in different organisms and correlation with proteins of established function [18].

By combining network analysis and structural bioinformatics of viral proteins with traditional phylogenetics, researchers can overcome the limitations of basic bioinformatics tools. This integration enhances and strengthens the ability to study RNA virus evolution, track their genetic changes, and improve predictions of future pandemic outbreaks. As bioinformatics continues to advance, these methods are certain to play a significant role in virology, structural biology, and public health management.

### 4.4. Implication of the Results and Summary

The reviewed articles highlight the importance of advanced bioinformatic tools, which play a crucial role in analyzing viral genotypes, codons, proteins, classification, and RNA virus evolution. The future development of this field, along with the integration of metagenomic findings with bioinformatic tools, can substantially improve our preparedness for emerging threats posed by new and evolving RNA viruses.

Future research directions, based on the conclusions drawn from this review, should focus on further developing advanced bioinformatics tools and integrating metagenomics with phylogenetic analysis to enhance our understanding of RNA virus evolutionary pathways. Another critical area involves studying the mechanisms of HGT and homologous RNA recombination, which enable the emergence of new and potentially more virulent strains. Additionally, deeper investigation into the effects of selective pressures and codon preferences on virus adaptation may pave the way for the development of personalized antiviral therapies. Finally, intensified efforts in identifying and classifying novel RNA viruses are essential to effectively monitor and mitigate potential pandemic threats.

### 4.5. Implication for Public Health and Vaccine Development

This review aimed to highlight which bioinformatics tools assist in genome analysis and phylogenetic analysis of RNA viruses. The information presented can reveal connections between a virus and its host or trace its evolutionary pathway, aiding in predicting the emergence of infectious strains and assessing the risk of potential pandemics. These aspects are crucial for public health, as they provide an opportunity to prepare adequately for a pandemic.

Furthermore, obtaining information about a potential infectious virus allows for its early detection, which can limit its spread and contain it at the source. Additionally, genome analysis of the virus brings us closer to developing vaccines against it. By understanding its sequence, it becomes possible to attempt the creation of a vaccine using the same virus but with reduced infectivity.

Phylogenetic trees play a crucial role in assessing pandemic risk by facilitating the analysis of evolutionary relationships among pathogenic viruses and identifying potential threats. In the article [19], it is demonstrated how phylogenetic analysis can contribute to forecasting and preparing for potential future pandemics. The study outlines several approaches for using phylogenetic trees in pandemic risk assessment:

- **Identification of Pathogen Origins**: Phylogenetic analysis enables us to uncover the evolutionary history and origins of pathogens, thereby elucidating how new viral strains spread within human populations. For example, the phylogenetic study of SARS-CoV-2 revealed its relatedness to bat coronaviruses, highlighting its zoonotic origin.
- **Monitoring Mutations and Adaptations**: Phylogenetic frameworks help track genetic changes in pathogens over time. Monitoring these changes is invaluable for identifying new viral properties, such as drug resistance or increased infectivity. For instance, the phylogenetic analysis of the spike protein in SARS-CoV-2 has uncovered variants with potentially higher transmissibility.
- **Assessment of Cross-Species Transmission Risk**: Phylogenetic studies assist in evaluating the likelihood of pathogens spreading between species. By comparing viral genomes from different hosts, researchers can determine a virus’s potential for interspecies transmission—a critical factor in assessing the risk of emerging zoonoses.
- **Planning Control and Vaccination Strategies**: Understanding the phylogenetic relationships among pathogen strains aids in the development of vaccines and control measures. Phylogenetic analyses indicate which strains are most prevalent or exhibit traits associated with increased infectivity, thereby guiding targeted preventive strategies.

In summary, phylogenetic trees provide invaluable insights into the evolution and dissemination of pathogens, making them essential tools for assessing pandemic risk and developing effective strategies for the prevention and control of infectious diseases [19].

## 5. Conclusions

The review and analysis of scientific articles allowed for the identification of several key conclusions regarding the use of bioinformatics tools for the phylogenetic analysis of RNA viruses. The first, and perhaps most groundbreaking, insight is the role of metagenomics as a game changer. This method has revolutionized the study of RNA viruses by enabling the discovery and classification of viruses directly from their natural environments, while also revealing the extensive level of gene transfer, including horizontal transfer.

Another significant observation is the impact of bioinformatics-driven research on public health and medicine. These tools facilitate the development of vaccines and personalized therapies against specific viruses by analyzing codon preferences and genomic structures.

However, there are notable challenges hindering further research into the phylogenetics and evolution of viruses. One major limitation is the current state of bioinformatics tools, which exhibit high specificity for certain viruses, sensitivity to data quality, and an inability to fully capture viral evolutionary pathways, such as homologous recombination or horizontal gene transfer. Additionally, the rapid and complex evolution and mutation of viruses pose significant challenges to existing bioinformatics tools, which must continuously evolve to keep pace with nature. 

## Figures and Tables

**Table 1:** Virus taxonomy tools, inspired by [3].

Software/Method	Input	Classification of …	Developed for …	Advantages	Limitations
APCluster	Pairwise distance matrix of reference and new viruses	All viruses in data set	Classification of intra-species (Rabies virus)	Effective foe intra-species virus analysis	Limited use foe distantly related viruses
CASTOR	New and reference genome sequences (CASTOR-predict) or genome sequences + classification (CASTOR-build)	New or all viruses	Classification of new viruses or build new classification	Limited use foe distantly related viruses	Lack standardization for different viruses groups
DeMARc	Multiple sequence alignment, distance matrix or phylogenetic tree of reference and new viruses	All viruses in data set	Large monophyletic groups of viruses	Suitable for evolutionary analysis	Less precise for small datasets
GRAViTy	New and reference genome sequences + reference classification	New viruses	Classification into existing taxa (family or higher)	Useful for classifying new viruses within known families	Not suitable for discovering new viruses
mPTP/VicTree	New and reference genome sequences + reference classification	New viruses	Viruses from a monophyletic group	Accurate analysis of monophyletic virus groups	Less effective for highly diverse viruses
MrBayes	Genomic sequences (nucleotide or protein) + Bayesian inference using MCMC	New or all viruses, but does not classify new viruses automatically	All organisms, especially useful for RNA and DNA viruses	Very accurate phylogenetic inference	Requires significant computing power and time
MyCoV	New and reference genome sequences + taxon identifier	New viruses	Coronavirus subgenus classification	Specialized tool for coronaviruses	Limited to a single virus group
PASC	New genome sequences + reference distance matrix + reference classification	New viruses	Fast web-based genome-wide analysis	High computational efficiency	Limited accuracy for complex phylogenies
PhyCLIP	Phylogenetic tree + distance threshold offset + false discovery rate for reference and new viruses	All viruses in data set	Intra-species (Influenza virus) classification	High precision for intra-species analysis	Not suitable for broad classification
PhyloPlace	New and reference genome sequences + reference classification	New viruses	HCV and HIV-1 subtypes	Well-suited for analyzing HIV and HCV subtypes	Limited application to other viruses
SDT	Genome or protein sequences of new and reference sequences + reference classification	New viruses	New viruses from a monophyletic group	Effective for closely related viruses	May be unreliable for highly diverse groups
VConTACT	New and reference genome sequences + reference classification	New viruses	Archaeal and bacterial viruses	Specializes in bacteriophage classification	Not applicable to eukaryotic viruses
VICTOR	New and reference genome sequences + reference classification	All viruses in data set	Archaeal and prokaryotic viruses	Versatile for prokaryotic virus classification	Less precise for eukaryotic viruses
VIRIDIC	New and reference genome sequences + distance thresholds	All viruses in data set	Prokaryotic viruses	Effective for bacteriophage analysis	Limited to prokaryotic viruses

**DNA**: DeoxyriboNucleic Acid; **HCV**: Hepatitis C Virus; **HIV**: Human Immunodeficiency Virus; **MCMC**: Markov Chain Monte Carlo; **RNA**: RiboNucleic Acid.

## Data Availability

The data and other materials used in this review are available online through several databases listed in the bibliography. Each part of the review includes citations referencing specific scientific articles. The sources provide detailed information needed to locate the referenced research papers. The results of this review have not been published anywhere else.

## Appendix A

This appendix outlines the process of searching for and selecting the articles that were chosen as the sources for this review. Additionally, it will describe the methods used to verify the credibility of these works, as well as the databases in which they were found. The entire process aimed to gather as much available information as possible to create this review. A key element was identifying relevant keywords and establishing appropriate criteria for filtering results in the databases. Furthermore, an important aspect was to find a suitable method for analyzing the selected scientific studies to obtain comprehensive information as accurately as possible. Part of the article selection process also involved determining the criteria for the relevance to the topic. Finally, this appendix will present the reasons and factors that influenced the selection of these works, along with an assessment of their significance and reliability.

### Appendix A.1. Eligibility Criteria

The selection of articles was focused on the number of citations by other scientists, how recent a given article is and whether the themes of the work fit the scope of this review. The attention was also drawn to where the article was originally published. The selected articles had to be relevant to the key concepts of interest in order to provide the necessary foundation for writing this paper. The main focus was on the genome of the virus, the bioinformatics tools that were used in the study and whether the scientific work draws attention to the phylogenetics of viruses. All of these factors contributed to the selection of the articles searched for and which papers will ultimately be used to prepare the review.

### Appendix A.2. Information Sources

The main source for article searches were the following databases: Google Scholar, PubMed, Web of Science, and ScienceDirect. The access to the full articles was received, where they could be seen in their original form (PDF), on the following websites: PMC (PubMed Central), ScienceDirect, International Society for Computational Biology (Oxford Academic) and MDPI. Each article was inspected many times and the works were initially searched in the last week of September 2024 and then repeatedly analyzed and reviewed in favor of writing this scientific review.

### Appendix A.3. Search Strategy

While searching scientific papers in databases, various filters and keywords were used to make it easier to find the right article to prepare for this review. The goal was to narrow down the list of searched articles with a few filters:

- Limiting the release date to 2010 for the latter, so that the information presented in the articles is up to date;
- Referencing not only articles but also books since the year of publication 1997;
- Number of citations.

In addition, the keywords used to find articles were RNA viruses, bioinformatics tools, and phylogenetic analysis.

### Appendix A.4. Selection Process

All studies were checked and analyzed by one person. The selected articles have been reviewed several times to determine if they meet the criteria to be used as sources for this paper. The methods used to decide whether a given study can be used was to find in them the appropriate data, information and results that fully represent the discussed problem.

### Appendix A.5. Data Collection Process

Data and information collection was carried out in selected articles by reading and highlighting the most important elements. In addition, while analyzing these reports, the focus was on creating notes from the article for an easier and more transparent way of writing the review paper.

### Appendix A.6. Data Items

The goal was to write down and describe the bioinformatics tools used during the research, to find information about the structure and analysis of the genetic material of the virus and how phylogenetic analysis was carried out, and thus to create a tree of phylogenetic RNA of the virus. It was essential to find information in the articles about the tools used for phylogenetic analysis. Most of the information on the concepts was similar and overlapped in most of the articles. Each selected study used different methods of phylogenetic analysis and various bioinformatics tools to analyze the RNA of the virus. This diversity helped to gain more knowledge on the topic. The articles focused on different species of viruses so that comparison of their genetic structures and finding similarities in evolution or searching for connections in phylogenetic trees were possible. The database of selected articles is extensive and full of diverse but common knowledge.

### Appendix A.7. Study Risk of Bias Assessment

The risk assessment of partiality in the articles used as sources for the paper was carried out by one person. The analysis was automated with the help of Artificial Intelligence tools. It was used to compile observations from the results and information from the article and also to compare the conclusions drawn by the reviewer. Each source article was selectively analyzed, which resulted in the information being successfully extracted. Each time the conclusions were rechecked and—when there was such need—the collected materials were updated. Also, in order to standardize the data for the scientific review, the information from the articles was compared with each other, in order to understand and confirm whether the research from different sources factually align with one another.

### Appendix A.8. Effect Measures

While working on this review, no laboratory or statistical tests were performed and therefore there is no way to give a measure of the effects of each result. It can be stated that the resulting review was created on the basis of the scientific literature, where the results already produced were confirmed and verified, so the purpose of this work was to find a common denominator which is information on the phylogenetic analysis of genetic material of RNA viral using bioinformatics tools. This article was based on the evaluation of other studies and taking a closer look at the most important information that led the reviewer to similar conclusions. It can be said that each result from one article was confirmed in the second article and it was empirically proven by the author, so it is impossible to clearly determine the measure of the effect.

### Appendix A.9. Synthesis Methods

While working on this review, there were several scientific articles selected as sources. In these scientific studies, there were many tables, diagrams or illustrations showing the results of the studies with their purpose being the facilitation of the presentation of the results and their analysis. Based on the information collected from the article, it could be seen that Metagenomics played an important role in the identification of new RNA groups of viruses. It allowed extensive research into the evolution of these viruses by sequencing the virus genome from different backgrounds and rearranging them using genetic similarity. The results of the studies conducted in these scientific works are presented in tables comparing all the features of the virus genome, while the phylogenetic trees are presented using diagrams showing the evolutionary relationships between the families of the analyzed strains. In a situation where the meta-analysis did not allow us to see the results of the research, it was replaced with narrative synthesis usage, the purpose of which was to find similarities and differences between the analyzed genomes. With the right amount of data from the meta-analysis in the articles, evolutionary trees were created, on the basis of which it was possible to come to conclusions faster. In laboratory studies, those with low sequence coverage were excluded during data collection to ensure reliable results using sensitivity analysis. This approach proved itself helpful as it was imperative in assessing the transmissions conducted over the RNA of viruses, combining knowledge in the field of genetics and bioinformatics.

### Appendix A.10. Reporting Bias Assessment

In order to avoid the risk of bias while writing this review, many articles were used as sources. Based on their analysis, it was possible to conclude which information was repeated and which was missing. In order to confront the information that appeared in only one article, the main goal was to find other scientific works on similar topics that explained the differences and results.

### Appendix A.11. Certainty Assessment

Confidence in the reliability of selected sources was based on the belief that the databases used to search for articles were credible because they had been verified many times. Moreover, the authors of these selected scientific studies are educated in their respective fields that are related to the issues raised in the review. That and the fact they hold scientific degrees makes them trustworthy.
